# The Utility of Pentraxin and Modified Prognostic Scales in Predicting Outcomes of Patients with End-Stage Heart Failure

**DOI:** 10.3390/jcm11092567

**Published:** 2022-05-04

**Authors:** Wioletta Szczurek-Wasilewicz, Michał Skrzypek, Ewa Romuk, Mariusz Gąsior, Bożena Szyguła-Jurkiewicz

**Affiliations:** 1 Silesian Center for Heart Diseases in Zabrze, 41-800 Zabrze, Poland; 2 Department of Biostatistics, School of Public Health in Bytom, Medical University of Silesia, 40-055 Katowice, Poland; mskrzypek@sum.edu.pl; 3 Department of Biochemistry, School of Medical Sciences in Zabrze, Medical University of Silesia, 40-055 Katowice, Poland; eromuk@gmail.com; 4 3rd Department of Cardiology, School of Medical Sciences in Zabrze, Medical University of Silesia, 40-055 Katowice, Poland; m.gasior@op.pl (M.G.); centrala4@wp.pl (B.S.-J.)

**Keywords:** pentraxin-3, heart failure, scales, risk stratification

## Abstract

Risk stratification is an important element of management in patients with heart failure (HF). We aimed to determine factors associated with predicting outcomes in end-stage HF patients listed for heart transplantation (HT), with particular emphasis placed on pentraxin-3 (PXT-3). In addition, we investigated whether the combination of PTX-3 with the Heart Failure Survival Score (HFSS), the Seattle Heart Failure Model (SHFM), or the Meta-Analysis Global Group in Chronic Heart Failure (MAGGIC) improved the prognostic strength of these scales in the study population. We conducted a prospective analysis of 343 outpatients with end-stage HF who accepted the HT waiting list between 2015 and 2018. HFSS, SHFM, and MAGGIC scores were calculated for all patients. PTX3 was measured by sandwich enzyme-linked immunosorbent assay with a commercially available kit. The endpoints were death, left ventricular assist device implantation, and HT during the one-year follow-up. The median age was 56 (50–60) years, and 86.6% were male. During the follow-up period, 173 patients reached the endpoint. Independent risk factors associated with outcomes were ischemic etiology of HF [HR 1.731 (1.227–2.441), *p* = 0.0018], mean arterial pressure (MAP) [1.026 (1.010–1.042), *p* = 0.0011], body mass index (BMI) [1.055 (1.014–1.098), *p* = 0.0083], sodium [1.056 [(1.007–1.109), *p* = 0.0244] PTX-3 [1.187 (1.126–1.251, *p* < 0.0001) and N-terminal pro-brain natriuretic peptide (NT-proBNP) [HR 1.004 (1.000–1.008), *p* = 0.0259]. The HFSS-PTX-3, SHFM-PTX-3 and MAGGIC-PTX-3 scores had significantly higher predictive power [AUC = 0.951, AUC = 0.973; AUC = 0.956, respectively] than original scores [AUC for HFSS = 0.8481, AUC for SHFM = 0.7976, AUC for MAGGIC = 0.7491]. Higher PTX-3 and NT-proBNP concentrations, lower sodium concentrations, lower MAP and BMI levels, and ischemic etiology of HF are associated with worse outcomes in patients with end-stage HF. The modified SHFM-PTX-3, HFSS-PTX-3, and MAGGIC-PTX-3 scores provide effective methods of assessing the outcomes in the analyzed group.

## 1. Introduction

Despite major drug and device therapy advances, the number of patients with end-stage heart failure (HF) requiring a heart transplant (HT) is gradually increasing [1,2]. The limited supply of donor hearts requires the need to search for new and simple tools that will facilitate patient allocation to the waiting list for HT. Over the years, many different prognostic models have been proposed to improve mortality risk assessment, optimize treatment and promote more effective use of therapy, each with its own set of advantages and limitations [3,4,5,6]. The widely used prognostic scale is the Heart Failure Survival Score (HFSS), which includes clinical variables and maximal oxygen uptake [3]. The Seattle Heart Failure Model (SHFM) offers a comprehensive risk assessment, including device therapy [4]. The comorbid conditions have been included in the Meta-Analysis Global Group in Chronic Heart Failure (MAGGIC) score, showing their significant utility in predicting patient outcomes [5]. Some biomarkers have also been shown to improve the diagnostic accuracy of preexisting models [7,8,9]. Despite these prognostic tools, there might still be room for further refinement of risk stratification through the use of new simple biomarkers and scales related to HF. Biomarkers that reflect the grade of inflammation and myocardial remodeling, such as pentraxin (PTX-3), may provide important information on risk stratification and monitoring HF therapy [10].

PTX-3 is a member of a superfamily of multimeric pattern-recognition proteins that play important roles at the interface of the innate immune response, inflammation, and extracellular matrix remodeling [11,12,13,14]. PTX-3 is produced by different vascular and inflammatory cells in response to primary inflammatory stimuli and might reflect local inflammatory status in the cardiovascular system [11,12,14]. The presence of PTX3 was detected in the myocardium in various pathological conditions, which was parallel to the observation of increased levels of PTX3 in plasma in patients with cardiovascular disorders [13]. However, the clinical significance of the plasma PTX3 levels in end-stage HF referred for HT has not been fully established.

Because of the potential relationship between PTX-3 and the pathogenesis of HF, we aimed to determine factors associated with predicting outcomes in end-stage HF patients listed for HT, with particular emphasis placed on PTX-3. In addition, we investigated whether the combination of PTX-3 with SHFM, HFSS, or MAGGIC score improves the prognostic strength of these scales in our study population.

## 2. Materials and Methods

We conducted a prospective analysis of 383 outpatients with end-stage HF who were referred to our center and underwent qualification for HT between 2015 and 2018. Patients with contraindications to HT were excluded from the study (*n* = 40). In the analyzed patients, medical history, anthropometric measurements, physical examinations, transthoracic echocardiographic measurements, ergospirometric exercise tests, right heart catheterization, and a panel of laboratory tests were performed. All patients received standard medical treatment, including angiotensin-converting-enzyme inhibitors/angiotensin II receptor blockers, mineralocorticoid receptor antagonists, and beta-blockers, at the maximum tolerated doses for at least 3 months prior to study inclusion.

The glomerular filtration rate was estimated according to the Modification of Diet in Renal Disease equation.

The study was approved by the Bioethical Committee of the Medical University of Silesia (specific ethics code—KNW/0022/KB1/88/15, date of approval: 7 July 2015). The study conformed to the principles outlined in the Declaration of Helsinki on the ethical principles for medical research involving human subjects. Written informed consent was obtained from all included patients.

### 2.1. Laboratory Measurements

Venous blood samples were obtained under stable and fasting conditions to measure the serum levels of the laboratory test panel. The complete blood count and hematologic parameters were determined using automated blood cell counters (Sysmex XS1000i and XE2100, Sysmex Corporation, Kobe, Japan). Liver and kidney function parameters, as well as cholesterol and albumin plasma levels, were measured with a COBAS Integra 800 analyzer (Roche Instrument Center AG, Rotkreuz, Switzerland). A highly sensitive latex-based immunoassay was used to detect plasma C-reactive protein (CRP) with a Cobas Integra 70 analyzer (Roche Diagnostics, Ltd., Rotkreuz, Switzerland). The plasma concentration of fibrinogen was measured using an STA Compact analyzer (Roche). The plasma concentration of N-terminal prohormone of brain natriuretic peptide (NT-proBNP) was measured with a commercially available kit from Roche Diagnostics (Mannheim, Germany) on an Elecsys 2010 analyzer. Human PTX3 was measured by sandwich enzyme-linked immunosorbent assay (ELISA) with a commercially available kit (Human PTX3 ELISA Kit, SunRedBio Technology Co, Ltd., Shanghai, China). The concentration of PTX3 was expressed as ng/mL. The sensitivity of the assay was 0.051 ng/mL. The assay range was 0.08 ng/mL–20 ng/mL. This ELISA test was performed using a BioTek Elx50 reader (BioTek Instruments Inc., Tecan Group, Männedorf, Switzerland).

### 2.2. Scales

Three HF prognostic scores were analyzed in the entire cohort:

- The HFSS score was calculated based on the following equation incorporating seven variables: ([0.0216 × resting heart rhythm] + [−0.0255 × mean arterial blood pressure (MAP)] + [−0.0464 × left ventricular ejection fraction (LVEF)] + [−0.0470 × serum sodium] + [−0.0546 × peak VO_2_ ] + [0.6083 × presence (1) or absence (0) of interventricular conduction defect (QRS duration ≥ 0.12 due to any cause)] + [0.6931 × presence (1) or absence (0) of ischemic cardiomyopathy]), as described previously [3].

SHFM was derived on the basis of the original risk factor coefficients as described by Levy et al. The SHFM includes 10 continuous variables (age, LVEF, New York Heart Association (NYHA) class, systolic blood pressure, diuretic dose adjusted for weight, lymphocyte count, hemoglobin, serum sodium, total cholesterol, and uric acid) and 10 categorical variables (gender, ischemic cardiomyopathy, use of device therapy (implantable cardioverter-defibrillator, cardiac resynchronization therapy), use of beta-blockers, angiotensin-converting enzyme inhibitor, angiotensin receptor blockers, potassium-sparing diuretic, statins, and allopurinol) in an equation that provides a continuous risk score for each patient [4].

- The MAGGIC score [5] was developed from 13 routinely available patient characteristics: (1) age, (2) sex, (3) body mass index (BMI), (4) systolic blood pressure, (5) creatinine concentration, (6) presence or absence of diabetes mellitus and (7) chronic obstructive pulmonary disease, (8) HF diagnosed in the last 18 months, (9) NYHA class, (10) LVEF, (11) current smoking status, (12) b-blockers, and (13) angiotensin-converting enzyme inhibitors or angiotensin receptor blockers. From 18 September 2013, the integer score increased by 2 if HF was diagnosed >18 months ago, which is reflected in our analysis.

To assess the ability of PTX-3 to improve the prognostic values of the scales, new combined scores were created. The scores for HFSS and PTX-3, MAGGIC and PTX-3, as well as for SHFM and PTX-3, were included in the Cox regression model as continuous variables, and each variable was multiplied by its corresponding β-coefficient. The final scores for new scales were calculated based on the following formulas:

HFSS-PTX-3 = 0.1595 ∗ PTX3 − 0.9743 ∗ HFSS

MAGGIC-PTX-3 = 0.2025 ∗ PTX + 0.0943 ∗ MAGGIC

SHFM-PTX-3 = 0.2014 ∗ PTX + 0.3757 ∗ SHFM

The raw score for HFSS-PTX-3 was multiplied by (−1) to achieve a positive value and facilitate the interpretation of the results.

### 2.3. Outcome Data

The composite outcome was represented by death, lvad left ventricular assist device (LVAD), implantation, and HT during the one-year follow-up. Follow-up was performed according to the local HF program, with regular physician’s office visits (every 6 months). The information about the one-year mortality was based on the data obtained from the national health care provider.

### 2.4. Statistical Analysis

The statistical analysis was performed using SAS software, version 9.4 (SAS Institute Inc., Cary, NC, USA). Categorical variables are presented as counts and percentages. Continuous variables were evaluated for normal distribution assumption using the Kolmogorov–Smirnov and Shapiro–Wilk tests and were reported as the mean plus standard deviation in brackets or the median with lower and upper quartiles. Differences between the study groups were assessed using Student’s t-test, the Mann–Whitney test, or the χ^2^ test. The prognostic utility of each score was quantified by the area under the ROC curve (AUC), sensitivity, specificity, negative predictive value (NPV), positive predictive value (PPV), negative likelihood ratio (LR-), positive likelihood ratio (LR+) and accuracy. Comparison between areas under the curve (AUCs) was achieved with the method used by DeLong et al. The differences between the AUC values were tested using the Hanley and McNeil method. The Spearman rank correlation test was used for correlation analysis. The tolerance and variance inflation factor were used to assess the correlation between explanatory variables and to assess multicollinearity. Schoenfeld residuals were used to check the proportional hazards assumption. Cox proportional hazard regression analysis was used to determine which variables were significantly related to the composite endpoint. Only variables with *p* values less than 0.20 in the univariable Cox regression analysis were entered into the multivariable Cox regression analysis. A *p* value ≤ 0.05 was considered statistically significant.

## 3. Results

The final study group consisted of 343 patients with end-stage HF awaiting HT classified into NYHA functional classes III and IV (87.2% and 12.8%, respectively) and profiles 4 to 6 according to the Interagency Registry for Mechanically Assisted Circulatory Support (INTERMACS) classification. The demographic and clinical characteristics of patients in the pooled population and divided into event and nonevent groups are presented in Table 1.

During the one-year follow-up, 109 (31.8%) deaths occurred, 35 (20.2%) patients underwent HTx, and 29 (8.5%) received LVAD.

**Figure 1:**
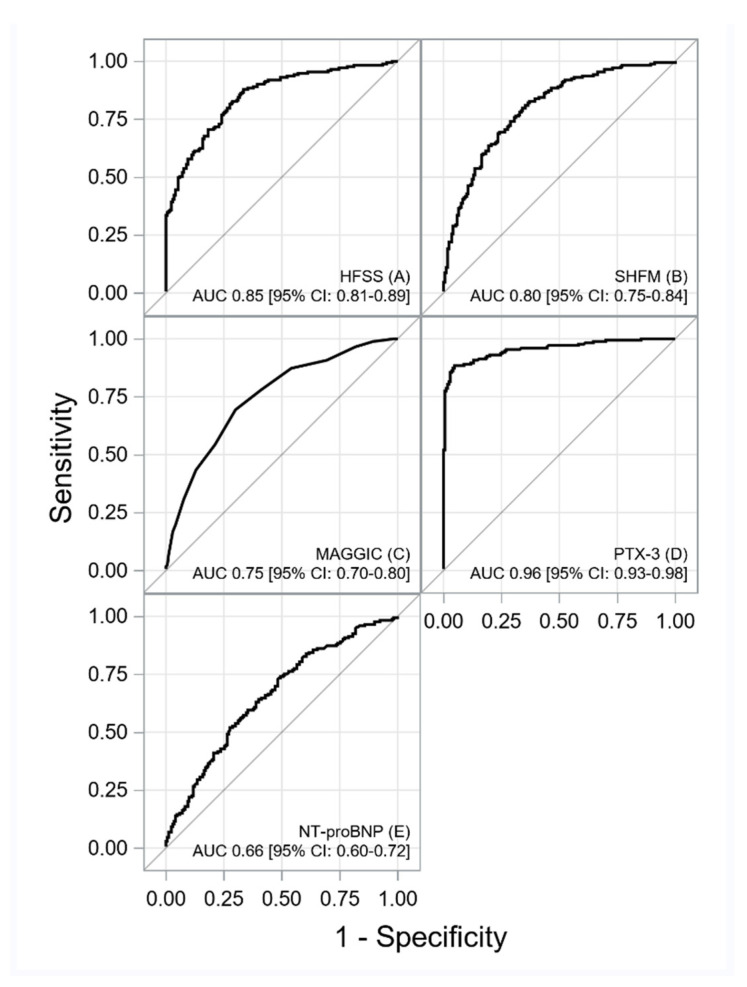
The ROC curves for HFSS (**A**), SHFM (**B**), MAGGIC (**C**), PTX-3 (**D**) and NT-proBNP (**E**). Abbreviations: AUC, are under the curve; HFSS— Heart Failure Survival Score; MAGGIC—Meta-Analysis Global Group in Chronic Heart Failure; NT-proBNP, N-terminal prohormone of brain natriuretic peptide; PTX-3, pentraxin-3; SHFM, Seattle Heart Failure Model; SHFM and MAGGIC scores revealed acceptable discrimination ability at 1 year of observation (AUC between 0.7 and 0.8), whereas the HFSS score showed good discrimination (AUC 0.85). PTX-3 displayed superior discriminative power against HFSS, MAGGIC, and SHFM scores for the composite endpoint.

**Figure 2:**
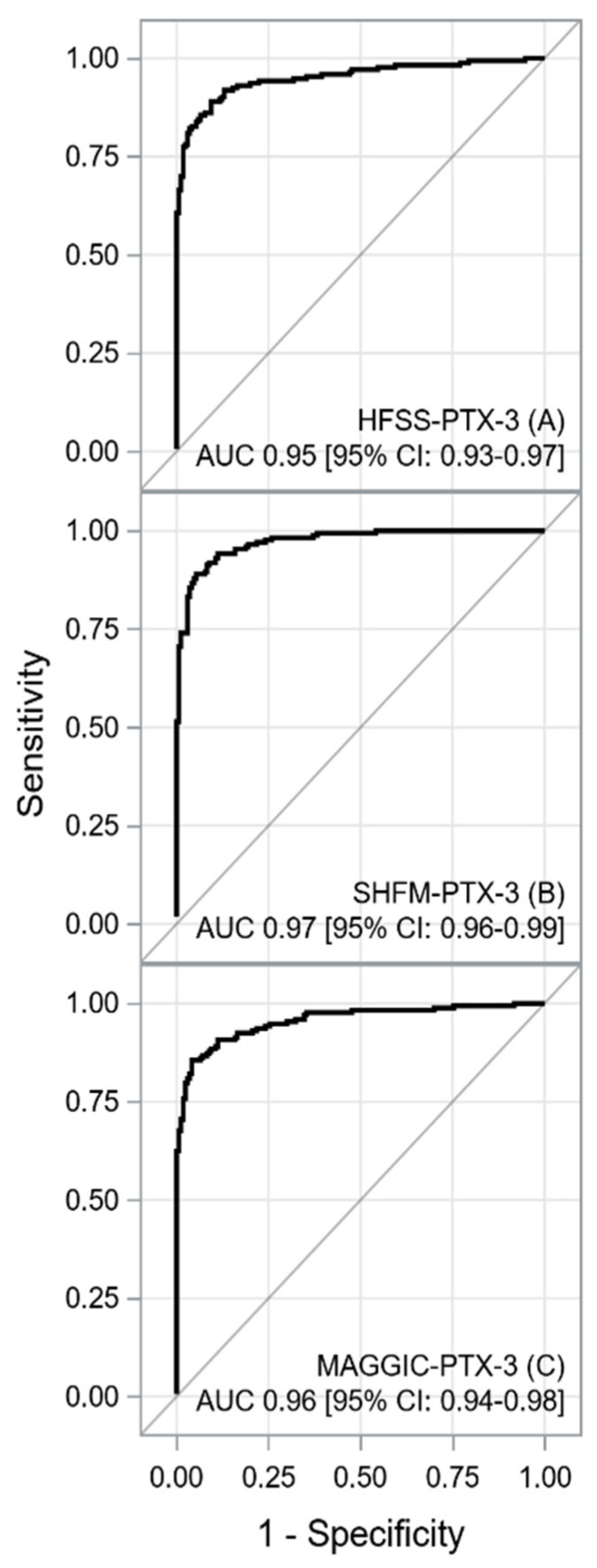
The ROC curves for HFSS + PTX-3 (**A**), SHFM + PTX-3 (**B**), MAGGIC + PTX-3 (**C**). Abbreviations: AUC, are under the curve; HFSS—Heart Failure Survival Score; MAGGIC—Meta-Analysis Global Group in Chronic Heart Failure; PTX-3, pentraxin-3; SHFM, Seattle Heart Failure Model. The HFSS-PTX-3, SHFM-PTX-3, and MAGGIC-PTX-3 scores generated excellent power to predict the composite endpoint (AUC > 0.90, *p* < 0.001). It is worth mentioning that combined scales reached high sensitivity, specificity, PPV, NPV, and accuracy and generated good results in terms of likelihood ratios. An improvement in AUC and *p* values for the composite endpoint was observed in the SHFM-PTX-3 score relative to those of individual components. In turn, HFSS-PTX-3 and MAGGIC-PTX-3 had a significant improvement in AUCs compared to the original scales. However, the prognostic power of HFSS-PTX-3 and MAGGIC-PTX-3 was comparable to that of PTX3.

The ROC curves for each score, NT-proBNP and PTX-3 are shown in Figure 1 and Figure 2.

A summary of the ROC curves analysis is shown in Table 2.

A comparison of the area under the ROC curves for the combined scales and their components is presented in Table 3.

The univariable and multivariable Cox proportional hazards analyses to predict composite endpoints for PTX3 and other variables are shown in Table 4. With the multivariable Cox proportional hazard analysis, ischemic etiology of HF, lower levels of mean arterial pressure MAP, BMI, and sodium, as well as higher levels of PTX-3 and NT-proBNP were independent predictors of the composite endpoint.

## 4. Discussion

This single-center study revealed an independent association between serum PTX-3 and worse outcomes in patients with advanced HF awaiting HT. PTX-3 serum concentrations allow for the accurate risk stratification of one-year outcomes in the analyzed group of patients. Previous studies have also confirmed the prognostic utility of PTX-3 in the assessment of outcomes in patients with chronic HF with reduced ejection fraction [12,15,16]. Kotooka et al. showed that higher plasma PTX3 levels are associated with a high risk of cardiac events in patients with HF. In addition, they also reported that PTX3 expression was higher in myocardial biopsy samples from HF patients compared to the control group [15]. In turn, Suzuki et al. demonstrated that plasma PTX3 concentration was increased in patients with HF compared to the control group and was also an independent predictor of cardiac events in HF patients [16]. Latili et al. also showed that baseline PTX3 levels and three-month changes in PTX3 levels were independently associated with worse outcomes in patients with chronic and stable HF. However, the authors demonstrated that after the addition of NT-proBNP to the prognostic model with PTX-3, only changes in PTX-3 concentrations were associated with outcomes [16].

HF is a systemic disorder that is associated with the activation of the inflammatory and immune systems [12,17,18]. Previous evidence has shown that activation of the inflammatory and immune systems may play an important role in HF [17,18,19]. From the pathophysiological point of view, PTX-3 can be associated with the development and progression of HF, especially due to its important regulatory role in inflammation, extracellular matrix organization, and remodeling [20,21,22]. PTX3 is produced by a variety of cell types, including monocytes/macrophages, vascular endothelial cells, vascular smooth muscle cells, adipocytes, fibroblasts, and dendritic cells [20,21,22]. Unlike CRP, which is synthesized in the liver and reflects systemic inflammation, PTX-3 is partially synthesized at the site of inflammation and released into the circulation, thus reflecting local inflammation in the cardiovascular system [16]. The main inducers of PTX-3 production are primary proinflammatory signals, such as interleukin-1β (IL-1β), TNFα, or bacterial molecules that engage Toll-like receptors (TLRs) [20]. In turn, elevated concentrations of these cytokines are observed in patients with HF in plasma and circulating leukocytes, as well as in the failed myocardium itself [17,21]. In addition, IL1β and TNFα are mediators involved in processes that lead to the remodeling of the heart, such as fibrosis and apoptosis [21]. The activation of the inflammatory system plays an important role in the pathogenesis of HF and is associated with an increase in plasma inflammatory cytokine levels, which stimulate the production of PTX-3. In turn, PTX-3 modulates inflammation in several cells, including endothelial cells, smooth muscle cells, and fibroblasts, and enhances further remodeling, which contributes to the intensification of the unfavorable cascade of changes in the heart muscle [12,15,20,21,22]. Another important property of PTX-3 is its ability to activate the classical complement activation pathway by binding the complement component C1q [23]. In turn, complement activation affects many processes related to the development and progression of HF, such as promotion of endothelial cell activation, monocyte infiltration into the extracellular matrix, and stimulation of cytokine release [24]. PTX3, by binding to the gamma Fc receptor, also influences the activation of MAP kinases, ERK1/2 and NF-κB proteins, which play an important role in heart remodeling [25,26].

Another interesting finding of our study is that PTX-3 may improve the prognostic value of recognized prognostic scales in patients with HF. The present study is the first to demonstrate that the modified SHFM-PTX-3, HFSS-PTX-3, and MAGGIC-PTX-3 scores provide effective methods of assessing the outcomes in patients with advanced HF awaiting HT. An improvement in prognostic power was observed in the SHFM-PTX-3 score relative to those of individual components. In turn, HFSS-PTX-3 and MAGGIC-PTX-3 had a significant improvement in prognostic power compared to the original scales.

This is an important finding because accurate risk stratification in patients with HF can prevent delays in the appropriate treatment of high-risk patients or the overtreatment of patients with low risk [27]. Sometimes there are difficulties in estimating the risk of death because of the multiplicity of risk factors related to HF and personal beliefs [28]. Therefore, objective risk scales are needed to assess the prognosis of patients with HF. There are many risk scales available; however, only two scales–the HFSS and SHFM–are included in the ISHLT guidelines for HT as prognostic tools in groups of patients with end-stage HF awaiting HT [28]. A relatively new scale, the MAGGIC, was originally developed in 2012 by Pocock et al. from a cohort of 39,372 patients with HF [5] and was confirmed in several external studies to have a discriminatory power ranging from 0.67–0.80 [5,7,29]. Our study showed for the first time that the prognostic power of the SHFM, HFSS, and MAGGIC scores were significantly improved when PTX-3 was added to the models. However, only in the case of SHFM-PTX-3 was better predictive power compared to both components observed. Some studies also showed that the prognostic power of HFSS, SHFM, and MAGGIC scores could be improved by adding other significant parameters associated with worse prognosis in patients with HF [5,7,9,30,31,32]. It seems that the modified risk scores may better stratify the outcomes by considering important risk factors in the current population of patients with HF and facilitate appropriate decisions regarding HF therapy.

Our study also confirms the importance of conventional HF risk factors. Lower sodium concentrations, higher NT-proBNP concentrations, lower BMI levels, lower MAP, and ischemic etiology of HF were also associated with an increased risk of worse outcomes during a one-year follow-up in the analyzed group of patients.

Lower serum sodium is an important and well-known factor of worse prognosis in patients with HF [4,33,34]. Moreover, sodium concentrations are also a component of some prognostic models in patients with HF [8,35]. Another factor related to a worse prognosis in our population, NT-proBNP, is one of the most widely researched and used biomarkers in everyday practice [8,34,36]. Many studies have confirmed the importance of NT-proBNP as an indicator of mortality and morbidity in various HF patient populations [8,36]. The inverse relationship between BMI and prognosis in HF is well known as the “obesity paradox”, in which a lower BMI is a factor in worse outcomes in HF [35,37]. Furthermore, BMI is one of the parameters of the MAGGIC score [5]. Similar paradoxical relationships are observed for blood pressure because lower SBP and MAP levels are associated with worse outcomes in HF [3,38,39]. Our results are completely in line with this. In addition, the MAP value is an important factor in the HFSS score, and SBP is included in the SHFM score [4]. The ischemic etiology of HF is also a well-known predictive factor of worse outcomes in patients with HF and has been widely discussed in the literature [4,40,41]. Moreover, ischemic etiology is a component of both the HFSS and the SHFM [3,4].

### Limitations

This single-center study has several limitations. Our study analyzed only the baseline PTX3 concentration at the time of inclusion in the study, while serial measurements of PTX-3 concentration over time might be more useful for evaluating one-year outcomes in ambulatory patients with HF. Furthermore, there was a lack of an independent validation cohort that could support our results. It is likely that if an independent validation cohort were used, the AUC for PTX-3 and other analyzed parameters would be lower. Although the size of the study group is relatively large for a single-center study, it can be considered small on the epidemiological scale. Considering the intrinsic limitations related to a single-center study and a small sample size, further multicenter studies with a large study population are necessary to confirm the clinical significance of PTX3 in the population of patients with HF.

## 5. Conclusions

In summary, our study showed that PTX-3 is a strong independent predictor of worse outcomes in end-stage HF patients awaiting HT. PTX-3 serum concentrations with excellent predictive power, sensitivity, and specificity allow for the accurate risk stratification of one-year outcomes in the analyzed group of patients. Furthermore, PTX-3 may improve the prognostic value of recognized scales, and the modified SHFM-PTX-3, HFSS-PTX-3, and MAGGIC-PTX-3 scores provide effective methods of assessing the outcomes in patients with advanced HF awaiting HT. Our study also confirms an independent association between conventional HF risk factors: higher NT-proBNP concentrations, lower sodium concentrations, lower MAP, lower BMI levels, ischemic etiology of HF, and worse outcomes in analyzed populations.

## Figures and Tables

**Table 1:** Baseline population characteristics and comparison between alive and events group.

	Overall Population N = 343 ^#^	Patients without Events N = 170	Patients with Events N = 173	*p*
Baseline data				
Age, years	56 (50–60)	56 (49–61)	56 (50–60)	0.7533
Male, *n* (%)	297 (86.6)	150 (88.2)	147 (85)	0.3751
Ischemic etiology of HF, *n* (%)	199 (58)	73 (42.9)	126 (72.8)	<0.0001 *
SBP, mmHg	102.00 (92.00–116.00)	113.00 (100.00–120.00)	98.00 (90.00–105.00)	<0.0001 *
MAP, mmHg	76.67 (71.67–85.33)	81.33 (76.00–90.00)	73.33 (68.67–78.33)	<0.0001 *
BMI, kg/m^2^	26.93 (23.85–30.08)	27.47 (24.49–31.21)	26.15 (23.25–29.05)	0.0002 *
NYHA III, *n* (%)	299 (87.2)	163 (95.9)	136 (78.6)	<0.0001 *
NYHA IV, *n* (%)	44 (12.8)	7 (4.1)	37 (21.4)	
Comorbidities				
Hypertension, *n* (%)	168 (49)	82 (48.2)	86 (49.7)	0.7846
Type 2 diabetes, *n* (%)	177 (51.6)	80 (47.1)	97 (56.1)	0.095
Persistent FA*,* *n* (%)	160 (46.6)	85 (50)	75 (43.4)	0.2173
COPD, *n* (%)	42 (12.2)	20 (11.8)	22 (12.7)	0.788
Laboratory parameters				
WBC, ×10^9^/L	7.18 (6.02–8.46)	6.96 (5.84–8.27)	7.33 (6.21–8.72)	0.1256
Lymphocytes, %	24.00 (17.70–30.06)	22.75 (17.80–28.60)	25.10 (17.70–32.50)	0.1179
Hemoglobin, mmol/L	8.80 (8.20–9.60)	8.80 (8.20–9.50)	8.90 (8.20–9.70)	0.4866
Creatinine, µmol/L	108.00 (93.00–126.00)	103.00 (88.00–121.00)	113.00 (102.00–134.00)	<0.0001 *
GFR, mL/min/1.73 m^2^	61.78 (51.63–75.73)	68.11 (55.49–81.65)	56.81 (50.15–68.78)	<0.0001 *
Platelets, ×10^9^/L	197.00 (172.00–228.00)	193.00 (171.00–220.00)	206.00 (175.00–237.00)	0.0317 *
Total bilirubin, µmol/L	18.40 (1220–24.10)	17.35 (11.30–21.90)	20.00 (13.40–25.90)	0.0021 *
Albumin, g/L	44.00 (41.00–46.00)	44.00 (42.00–46.00)	43.00 (41.00–46.00)	0.0445 *
Uric acid, µmol/L	441.00 (371.00–526.00)	403.50 (339.00–483.00)	470.00 (403.00–565.00)	<0.0001 *
Urea, µmol/L	8.10 (5.90–12.60)	7.35 (5.60–10.30)	8.90 (6.20–13.80)	0.0059 *
Sodium, mmol/L	139.00 (136.00–140.00)	140.00 (139.00–141.00)	137.00 (135.00–139.00)	<0.0001 *
Fibrinogen	379.00 (312.00–443.00)	363.50 (296.00–424.00)	396.00 (330.50–483.50)	0.001 *
AST, U/L	26.00 (20.00–31.00)	26.00 (20.00–31.00)	25.00 (20.00–33.00)	0.6419
ALT, U/L	22.00 (16.00–32.00)	23.00 (17.00–33.00)	21.00 (16.00–30.00)	0.1087
ALP, U/L	78.00 (62.00–102.00)	75.50 (58.00–101.00)	81.00 (65.00–102.00)	0.0642
GGTP, U/L	73.00 (35.00–125.00)	64.50 (34.00–111.00)	84.00 (36.00–137.00)	0.0256 *
Cholesterol, mmol/L	4.54 (4.16–5.00)	4.43 (4.02–4.86)	4.62 (4.22–5.15)	0.0019 *
LDL, mmol/L	2.05 (1.58–2.83)	2.04 (1.55–2.73)	2.06 (1.61–2.93)	0.43
hs-CRP, mg/L	3.40 (1.64–8.75)	2.75 (1.50–5.42)	4.52 (2.06–10.75)	0.0003 *
HBA1c, %	5.80 (5.40–6.30)	5.80 (5.40–6.30)	5.80 (5.30–6.30)	0.2926
NT-proBNP, pg/mL	4334.00 (1965.00–7102.00)	3023.00 (1743.00–6101.00)	5539.00 (2916.00–8310.00)	<0.0001 *
Pentraxin-3, ng/mL	3.65 (2.57–6.23)	2.58 (2.10–3.25)	6.22 (5.12–8.66)	<0.0001 *
Haemodynamic parameters				
mPAP, mmHg	23.00 (17.00–30.00)	23.50 (17.00–30.00)	22.00 (18.00–30.00)	0.7619
CI, l/min/m^2^	1.84 (1.72–1.94)	1.83 (1.70–1.94)	1.84 (1.72–1.95)	0.4959
TPG, mmHg	8.00 (6.00–10.00)	8.00 (6.00–10.00)	8.00 (6.00–10.50)	0.9387
PVR, Wood units	1.97 (1.47–2.36)	1.99 (1.56–2.35)	1.95 (1.41–2.35)	0.6186
Echocardiographic parameters				
LA, mm	52.00 (48.00–56.00)	52.00 (46.00–56.00)	53.00 (49.00–57.00)	0.0708
RVEDd, mm	34.00 (30.00–40.00)	33.00 (30.00–40.00)	34.00 (31.00–41.00)	0.065
LVEDd, mm	73.00 (68.00–80.00)	73.00 (68.00–80.00)	73.00 (69.00–81.00)	0.2354
LVEF, %	18.00 (15.00–20.00)	19.00 (16.00–21.00)	17.00 (15.00–20.00)	<0.0001 *
Treatment				
B-blockers, *n* (%)	320 (93.3)	161 (94.7)	159 (91.9)	0.7176
ACEI, *n* (%)	244 (71.1)	127 (74.7)	117 (67.6)	0.1482
ARB, *n* (%)	74 (21.6)	31 (18.2)	43 (24.9)	0.1361
Loop diuretics, *n* (%)	343 (100)	170 (100)	173 (100)	
MRA, *n* (%)	322 (93.9)	159 (93.5)	163 (94.2)	0.7898
Digoxin, *n* (%)	102 (29.7)	51 (30)	33 (19.1)	0.9161
Ivabradine, *n* (%)	63 (18.4)	30 (17.8)	33 (19.1)	0.7522
Statin, *n* (%)	261 (76.1)	135 (79.4)	126 (72.8)	0.1532
Coumarin derivatives, *n* (%)	186 (54.2)	93 (54.7)	93 (53.8)	0.86
Acetylsalicylic acid, *n* (%)	124 (36.2)	59 (34.7)	65 (37.6)	0.5806
Allopurinol, (*n*%)	163 (47.5)	76 (44.7)	87 (50.3)	0.3006
ICD *n* (%)	201 (58.6)	97 (57.1)	104 (60.1)	0.5655
CRT-D *n* (%)	142 (41.4)	73 (42.9)	69 (39.9)	
Other parameter				
VO_2_ max, mL/kg/min	10.80 (10.00–11.50)	10.90 (10.20–11.60)	10.70 (9.70–11.30)	0.0216 *
Current smoker, %	40 (11.7)	13 (7.6)	27 (15.6)	0.0217 *
QRS > 0.12 s	136 (39.7)	52 (30.6)	84 (48.6)	0.0007 *
Scales				
MAGGIC score	26.00 (24.00–29.00)	25.00 (23.00–27.00)	28.00 (26.00–30.00)	<0.0001 *
SHFM score	0.43 (−0.004–0.907)	0.10 (−0.25–0.46)	0.73 (0.39–1.26)	<0.0001 *
HFSS score	7.65 (7.22–8.20)	8.08 (0.52)	7.31 (0.54)	<0.0001 *

^#^ Data are presented as medians (25th–75th percentile) or numbers (percentage) of patients. * *p* < 0.05 (statistically significant). Abbreviations: ACEI, angiotensin-converting-enzyme inhibitor; ALP, alkaline phosphatase; ALT, alanine aminotransferase; ARB, angiotensin II receptor blocker; AST, aspartate aminotransferase; BMI, body mass index; CI, cardiac index; COPD—chronic obstructive pulmonary disease; CRT-D, cardiac resynchronization therapy-defibrillator; FA, atrial fibrillation; FEV1, forced expiratory volume in 1 s; FVC, forced vital capacity; GFR, glomerular filtration rate; GGTP, gamma-glutamyl transpeptidase; HBA1c, glycated hemoglobin; HF, heart failure; ; HFSS—Heart Failure Survival Score; HR, heart-rhythm; hs-CRP, high-sensitivity C-reactive protein; ICD, implantable cardioverter-defibrillator; LA, left atrium; LDL, low density lipoprotein; ; LVEDd, left ventricular end-diastolic dimension; LVEF, left ventricular ejection fraction; MAGGIC—Meta-Analysis Global Group in Chronic Heart Failure; MAP, mean arterial pressure; mPAP, mean pulmonary artery pressure; MRA, mineralocorticoid receptor antagonists; NT-proBNP, N-terminal prohormone of brain natriuretic peptide; PH, pulmonary hypertension; PVR, pulmonary vascular resistance; RVEDd, right ventricular end-diastolic dimension; SBP, systolic blood pressure; SHFM, Seattle Heart Failure Model; sPAP, systolic pulmonary artery pressure; TPG, transpulmonary gradient; Vo_2_ max, maximal oxygen uptake; WBC, white blood cells.

**Table 2:** A summary of ROC curves analysis for analyzed parameters.

	AUC [±95 CI]	*p*	Cut-off	Sensitivity [±95 CI]	Specificity [±95 CI]	PPV [±95 CI]	NPV [±95 CI]	LR+ [±95 CI]	LR- [±95 CI]	Accuracy
HFSS	0.8481 [0.8079–0.8883]	<0.0001	<7.86	0.88 [0.82–0.92]	0.66 [0.59–0.74]	0.73 [0.66–0.79]	0.84 [0.77–0.90]	2.62 [2.04–3.20]	0.18 [0.11–0.26]	0.77 [0.72–0.82]
SHFM	0.7976 [0.7510–0.8442]	<0.0001	≥0.299	0.80 [0.73–0.85]	0.66 [0.58–0.73]	0.70 [0.63–0.77]	0.76 [0.68–0.83]	2.34 [1.82–2.86]	0.31 [0.21–0.40]	0.73 [0.68–0.78]
MAGGIC	0.7491 [0.6979–0.8003]	<0.0001	≥27	0.69 [0.62–0.76]	0.70 [0.63–0.77]	0.70 [0.63–0.77]	0.69 [0.62–0.76]	2.31 [1.73–2.89]	0.44 [0.33–0.55]	0.70 [0.65–0.75]
PTX-3	0.9558 [0.9345–0.9772]	<0.0001	≥3.926	0.88 [0.83–0.93]	0.95 [0.91–0.98]	0.95 [0.91–0.98]	0.89 [0.84–0.93]	18.79 [5.95–31.63]	0.12 [0.07–0.17]	0.92 [0.88–0.94]
NT-proBNP	0.6598 [0.6024–0.7171]	<0.0001	≥3136	0.73 [0.66–0.79]	0.52 [0.44–0.59]	0.61 [0.54–0.67]	0.65 [0.56–0.73]	1.51 [1.24–1.78]	0.52 [0.37–0.67]	0.62 [0.57–0.68]
HFSS+ PTX-3	0.9508 [0.9277–0.9838]	<0.0001	<6.772	0.89 [0.83–0.93]	0.91 [0.85–0.95]	0.91 [0.85–0.94]	0.89 [0.83–0.93]	9.46 [4.99–0.1393]	0.12 [0.07–0.17]	0.90 [0.86–0.93]
SHFM+ PTX3	0.9727 [0.9588–0.9867]	<0.0001	≥1.062	0.89 [0.83–0.93]	0.95 [0.90–0.98]	0.94 [0.90–0.97]	0.89 [0.84–0.94]	16.81 [6.01–27.61]	0.12 [0.07–0.17]	0.92 [0.88–0.95]
MAGGIC+ PTX-3	0.9562 [0.9354–0.9770]	<0.0001	≥3.388	0.86 [0.79–0.90]	0.96 [0.92–0.98]	0.95 [0.91–0.98]	0.87 [0.81–0.91]	20.78 [5.55–36.00]	0.15 [0.10–0.21]	0.91 [0.87–0.94]

Abbreviations: see Table 1, AUC, area under the curve; LR*−*, negative likelihood ratio; LR*+*, positive likelihood ratio; NPV, negative predictive value; PPV, positive predictive value.

**Table 3:** A comparison of the area under the ROC curves for the combined scales and their components.

	HFSS-PTX-3, AUC [±95 CI] ^1^	*p*
HFSS, AUC [±95 CI]	0.1027 [0.0754–0.1299]	<0.0001
PTX-3, AUC [±95 CI]	−0.0051[−0.0285–0.0184]	0.6720
	SHFM-PTX-3, AUC [±95 CI]	*p*
SHFM, AUC [±95 CI]	0.1751 [0.1320–0.2183]	<0.0001
PTX-3, AUC [±95 CI]	0.0169 [0.0014–0.0324]	0.0330
	MAGGIC-PTX-3, AUC [±95 CI]	*p*
MAGGIC, AUC [±95 CI]	0.2071 [0.1637–0.2505]	<0.0001
PTX-3, AUC [±95 CI]	0.0004 [−0.0186–0.0194]	0.9693

Abbreviations: see Table 1; AUC, area under the curve. ^1^ The difference between AUCs.

**Table 4:** Univariable and multivariable Cox proportional hazard analysis of factors associated with events.

Parameter	Univariable Data		Multivariable Data	
Etiology of HF	2.075 [1.477–2.914]	<0.0001	1.731 [1.227–2.441]	0.0018
MAP ^(−)^	1.044 [1.030–1.059]	<0.0001	1.026 [1.010–1.042]	0.0011
BMI ^(−)^	1.073 [1.034–1.114]	0.0002	1.055 [1.014–1.098]	0.0083
NYHA IV ^(+)^	1.990 [1.354–2.924]	0.0005		
Creatinine ^(+)^	1.012 [1.006–1.018]	0.0001		
Bilirubin ^(+)^	1.016 [1.005–1.027]	0.0030		
Uric acid ^(+)^	1.002 [1.001–1.003]	0.0029		
hs-CRP ^(+)^	1.040 [1.019–1.061]	0.0001		
Na ^(−)^	1.157 [1.107–1.209]	<0.0001	1.056 [1.007–1.109]	0.0244
GGTP ^(+)^	1.002 [1.000–1.004]	0.0382		
Cholesterol ^(+)^	1.304 [1.087–1.059]	<0.0001		
PTX-3 ^(+)^	1.268 [1.212–1.326]	<0.0001	1.187 [1.126–1.251]	<0.0001
NT-proBNP ^(a)^	1.007 [1.004–1.010]	<0.0001	1.004 [1.000–1.008]	0.0259

(+) per one unit increase; (−) per one unit decrease; ^a^ per 100 units increase. Abbreviations: see Table 1, HR, hazard ratio; CI, confidence intervals.

## Data Availability

The data presented in this study are available on request from the corresponding author.
